# Diagnostic pitfall of a rare variant of angiomyolipoma, epithelioid angiomyolipoma - a case report

**DOI:** 10.11604/pamj.2020.37.210.26269

**Published:** 2020-11-02

**Authors:** Michael Leonard Anthony, Prashant Durgapal, Prashant Joshi, Ankur Mittal, Rishabh Sahai, Sanjeev Kishore, Ashok Singh

**Affiliations:** 1 Department of Pathology and Laboratory Medicine, All India Institute of Medical Sciences, Rishikesh, India,; 2 Department of Urology, All India Institute of Medical Sciences, Rishikesh, India

**Keywords:** Epithelioid cells, perivascular, PEComa, renal cell carcinoma

## Abstract

Angiomyolipoma of the kidney is a common benign mesenchymal neoplasm of kidney. A rare variant, epithelioid angiomyolipoma, however, may show malignant behavior. We report a case of epithelioid angiomyolipoma in a patient not having tuberous sclerosis which was initially misdiagnosed as renal cell carcinoma. A 39-year-old woman presented with a history of flank pain. Ultrasonography revealed a left renal mass. Contrast-enhanced computed tomography (CECT) abdomen revealed mass involving hilum of the kidney. On core biopsy a possibility of renal cell carcinoma was suggested. The patient underwent radical nephrectomy. After immunohistochemical analysis, a final diagnosis of epithelioid angiomyolipoma was made. Renal epithelioid angiomyolipoma without adipocytic component is extremely rare. It is pivotal to keep a possibility of epithelioid angiomyolipoma whenever an epithelioid renal tumor is encountered showing marked pleomorphism and mitosis. The use of melanocytic markers and specific markers of renal cell carcinoma will aid the diagnosis.

## Introduction

Originally believed to be a hamartoma, angiomyolipomas are common benign mesenchymal tumors occurring in the kidney. They belong to the PEComa group of tumors derived from perivascular epithelioid cells [1]. Epithelioid angiomyolipoma is a rare variant that accounts for 4.6% of all resected angiomyolipomas [2]. It can be seen in association with tuberous sclerosis as well as sporadic [3]. Due to varied histomorphology of tumor cells and the presence of bizarre and multinucleated cells, it may be misdiagnosed as a variety of malignant neoplasms including renal cell carcinoma [4]. It warrants due attention especially in core biopsies where a misdiagnosis of an aggressive tumor will lead to radical management. Treatment of epithelioid angiomyolipoma depends on the size of the lesion. Large tumors are excised with or without chemotherapy [5]. The potential complication of these tumors includes retroperitoneal hemorrhage [5]. We report a case of epithelioid angiomyolipoma in a patient without signs of tuberous sclerosis which was misdiagnosed as renal cell carcinoma on core biopsy.

## Patient and observation

**Figure 1:**
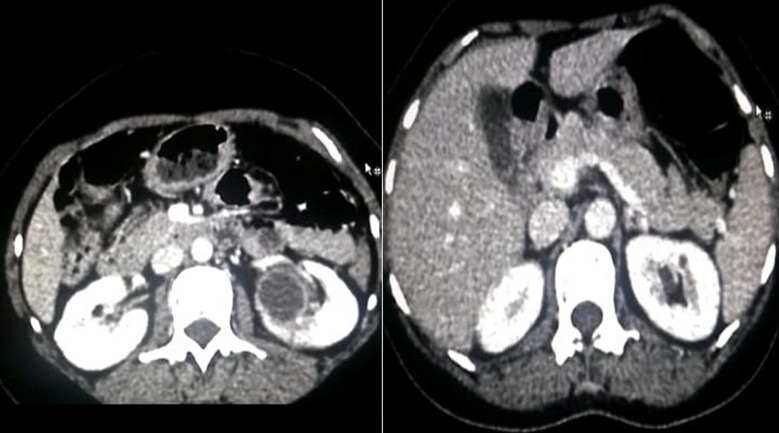
CECT images of the lesion involving left kidney; a large well-defined mass is seen in left kidney which was impressing upon and displacing renal vessels

**Figure 2:**
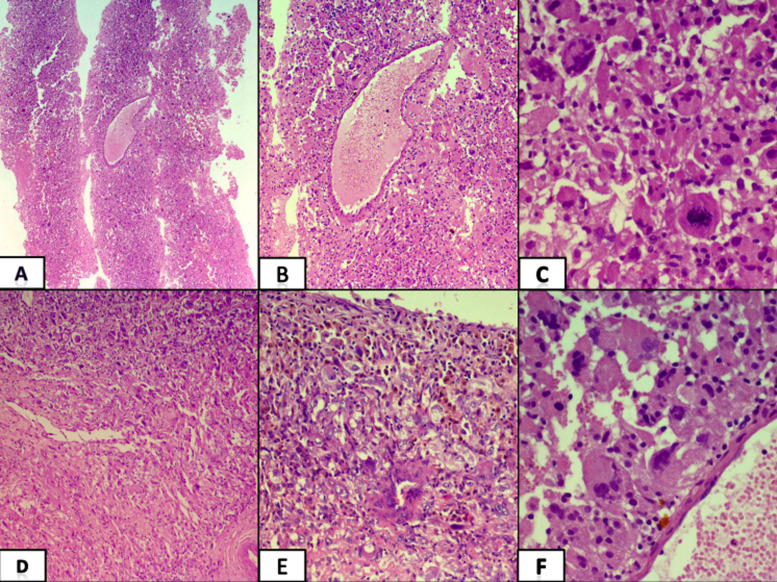
A-C) histopathology of core biopsy specimen; A) tumor cells in sheets (H&E X 40); B) peri vascular arrangement of tumor cells (H&E X 100); C) tumor cells showing marked pleomorphism, irregular nuclear contours and moderate cytoplasm with interspersed giant cells (H&E X 400); D-F) resection specimen of the tumor with similar morphology; numerous hemosiderin laden macrophages are also noted signifying old hemorrhage (D- H&E X 100, E- H&E X 200, F- H&E X 400)

A 39-year-old woman presented with a history of flank pain for 2 years without any associated complaints. Physical and systemic examination did not reveal significant findings. Routine investigations including complete blood count, renal function tests and liver function tests were within normal limits. Ultrasonography revealed a left renal lesion which was hypoechoic and involving hilum of the kidney. It was followed up with a contrast-enhanced computed tomography (CT) scan which showed a large well-defined mass measuring 45 x 42 x 39 mm in the left kidney which was impressing upon and displacing renal vessels (Figure 1). A core biopsy was done. Histopathological examination showed linear tissue cores infiltrated by tumor cells in sheets and arranged in a perivascular pattern. The tumor cells were round to polygonal showing marked nuclear pleomorphism, irregular nuclear contours, coarse chromatin with prominent nucleoli and a moderate amount of cytoplasm. Few cells showed eccentric nuclei along with few bizarre multinucleated giant cells (Figure 2). Immunohistochemical analysis demonstrated focally positive RCC antigen and CD 10 in tumor cells and a diagnosis of renal cell carcinoma was suggested.

**Figure 3:**
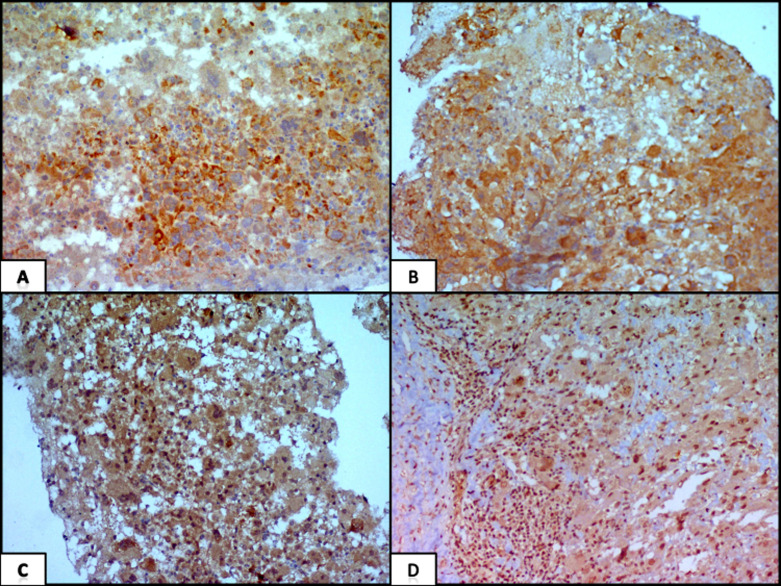
A-D) immunohistochemical markers; A) focal membranous positivity of CD 10; B) focal membranous positivity for RCC antigen; C) diffuse cytoplasmic and membranous positivity for melan; A, D) retained nuclear expression of INI-1 (IHC X 100)

The patient underwent radical nephrectomy. Gross examination of the kidney showed a well circumscribed lesion involving the middle one-third of the kidney measuring 4.4 x 4 x 3 cm. The cut surface of the lesion was yellow, friable with areas of necrosis. Histopathological examination revealed a similar morphology showing highly pleiomorphic tumor cells arranged in sheets along with numerous multinucleated giant cells. Many hemosiderin-laden macrophages were present along with areas of necrosis. Differential diagnoses considered were renal cell carcinoma with sarcomatoid features, vascular tumor, choriocarcinoma, epithelioid angiomyolipoma and rhabdoid tumor of kidney. Further immunohistochemical panels showed tumor cells were positive for HMB45, melan A, smooth muscle actin, focally positive for RCC antigen and CD 10 and negative for PanCk, CK 7, CEA, inhibin, calretinin, CD34, CD 31, vimentin and beta HCG. The immunoexpression of INI-1 was retained (Figure 3). A final diagnosis of epithelioid angiomyolipoma was made.

## Discussion

Renal epithelioid angiomyolipoma without adipocytic component is extremely rare accounting for 1% of renal angiomyolipomas [1]. Histological diagnosis of epithelioid angiomyolipoma requires more than 80% epithelioid cells [1]. Diagnosis remains challenging as histomorphology may mimic benign as well as malignant neoplasms. One of the close mimics is renal cell carcinoma with sarcomatoid features. The presence of interspersed spindled cells along with numerous bizarre giant cells further adds to the diagnostic dilemma. A core biopsy sample is misleading due to limited sampling. Immunohistochemistry is essential for the diagnosis of this entity. In the present case, vascular tumors were ruled out as tumor cells were negative for CD31 and CD34. Beta HCG, inhibin, calretinin was used to rule out choriocarcinoma and adrenocortical carcinoma.

INI-1 was done to rule out the rhabdoid tumor of kidney. Markers HMB 45, melan A are positive while cytokeratin and vimentin are negative which are important to differentiate from renal cell carcinoma [3]. However, these markers may be focally positive adding to the dilemma as in our case where focal positivity of markers present in renal cell carcinoma led to misdiagnosis of renal cell carcinoma. It is a potential diagnostic pitfall even after the use of immunohistochemistry. Two important markers to resolve this dilemma are PAX 8 and cathepsin K. Angiomyolipomas are negative for PAX 8 in contrast to renal cell carcinomas. Cathepsin K is useful in cases where HMB 45 and melan A are focally positive. Studies have shown that the majority of angiomyolipomas are diffusely positive for cathepsin K [6]. A literature search revealed few case reports where angiomyolipoma was misdiagnosed as renal cell carcinoma [7-9].

It is essential to differentiate it from renal cell carcinoma as epithelioid angiomyolipomas less than 4cm in size are treated conservatively. tumors larger than 4cm carries a fatal risk of hemorrhage as was in our case which showed foci of old hemorrhage in the tumor [4,10]. Radical surgery is done for such cases with or without chemotherapy with mTOR inhibitors [10]. Malignant behavior has been reported for epithelioid angiomyolipoma however predictors for malignant behavior are not well established. Size more than 7cm, mitosis and necrosis are few predictors of malignant behavior according to studies [4].

## Conclusion

It is pivotal to keep a possibility of epithelioid angiomyolipoma whenever an epithelioid renal tumor is encountered showing marked pleomorphism and mitosis. Use of melanocytic markers HMB 45, melan A, cathepsin K along with PAX 8, cytokeratin, vimentin is recommended in the immunohistochemical panels on core biopsies. Thorough sampling is necessary whenever a resection specimen is examined.
